# Supplementary material for: An Analysis of the Intestinal Microbiome Combined with Metabolomics to Explore the Mechanism of How Jasmine Tea Improves Depression in CUMS-Treated Rats
Source: Foods. 2024 Aug 22;13(16):2636. doi: 10.3390/foods13162636 (PMC11353544; doi:10.3390/foods13162636)
Supplement: Supplementary file 1 [file foods-13-02636-s001.zip › foods-3089781-supplementary.pdf]

Supplement materials

Table S1 The differential metabolites between the control and model group

Name	Formula	Molecular Weight	RT [min]	m/z	VIP	P-value	Trend
PC (18:5e/22:6)	$C_{48}H_{76}NO_7P$	809.521	16.561	810.527	2.246	0.000	Down
PC (16:1e/3:0)	$C_{27}H_{54}NO_7P$	535.362	14.979	536.369	2.061	0.001	Down
PC (14:0e/3:0)	$C_{25}H_{52}NO_7P$	509.347	15.002	510.355	2.046	0.001	Down
Tetrahydro cortisone	$C_{21}H_{32}O_5$	346.213	12.642	347.220	1.961	0.001	Down
2-[(3S)-1-(Cyclohexylmethyl)-3-pyrrolidinyl]-5-methyl-1H-benzimidazole	$C_{19}H_{27}N_3$	319.198	7.295	320.205	2.028	0.001	Down
1,4-Dihydro-1-Methyl-4-Oxo-3-Pyridinecarboxamide	$C_7H_8N_2O_2$	152.058	2.841	153.066	1.984	0.001	Down
α-Linolenic acid	$C_{18}H_{30}O_2$	278.223	16.871	279.230	1.949	0.002	Down
DAG (18:1/18:2)	$C_{39}H_{70}O_5$	635.547	16.014	636.554	1.962	0.002	Down
2-Oxindole	C_8H_7NO	133.053	8.763	134.060	1.899	0.004	Down
PC (18:2e/2:0)	$C_{28}H_{54}NO_7P$	547.362	14.881	548.369	1.807	0.005	Down
PC (15:0/16:0)	$C_{39}H_{78}NO_8P$	719.544	14.888	720.552	1.767	0.006	Down
LPE 17:0	$C_{22}H_{46}NO_7P$	467.300	14.823	468.307	1.848	0.007	Down
PC (19:2/22:6)	$C_{49}H_{82}NO_8P$	843.568	15.585	844.575	1.768	0.008	Down
SM (d14:3/22:1)	$C_{41}H_{77}N_2O_6P$	362.275	15.007	725.557	1.729	0.009	Down
MAG (18:2)	$C_{21}H_{38}O_4$	354.275	13.789	355.282	1.763	0.010	Down
Allolithocholic acid	$C_{24}H_{40}O_3$	376.296	14.660	377.303	1.894	0.011	Down
PC (16:0/17:2)	$C_{41}H_{78}NO_8P$	743.545	15.567	744.552	1.713	0.011	Down
O-7460	$C_{25}H_{48}FO_5P$	500.310	14.071	501.318	1.678	0.014	Down
PC (14:0e/22:6)	$C_{44}H_{78}NO_7P$	763.546	16.122	764.554	1.732	0.014	Down
3,14-dihydro-15-keto-tetranor Prostaglandin E2	$C_{16}H_{26}O_5$	298.177	11.086	299.184	1.659	0.017	Down
Palmitoyle thanolamide	$C_{18}H_{37}NO_2$	299.281	13.265	300.288	1.619	0.018	Down
PC (18:2e/16:0)	$C_{42}H_{82}NO_7P$	743.581	14.644	744.588	1.626	0.019	Down
Creatine	$C_4H_9N_3O_2$	131.069	1.282	132.076	1.673	0.019	Down

PC (14:1e/20:1)	$C_{42}H_{82}NO_7P$	743.582	15.220	744.588	1.613	0.019	Down
PC (16:0e/22:4)	$C_{46}H_{86}NO_7P$	795.614	14.555	796.621	1.682	0.020	Down
LPC 16:2	$C_{24}H_{46}NO_7P$	491.300	13.904	492.307	1.659	0.020	Down
4-(3,4-dihydro-2H-1,5-benzodiazepine-7-arylamino)-4-oxobutanoic acid	$C_{13}H_{15}NO_5$	247.080	1.153	248.088	1.650	0.021	Down
7-Ketolithocholic acid	$C_{24}H_{38}O_4$	390.276	13.139	391.283	1.563	0.024	Down
2-Arachidonoyl glycerol	$C_{23}H_{38}O_4$	360.265	14.400	361.272	1.604	0.024	Down
3-hydroxy-3-methyl pentane dioic acid	$C_6H_{10}O_5$	179.080	6.080	180.087	1.615	0.025	Down
PC (18:1/18:1)	$C_{44}H_{84}NO_8P$	785.590	16.648	786.598	1.500	0.027	Down
Cortisol	$C_{21}H_{30}O_5$	362.207	10.939	363.215	1.521	0.027	Down
SM (d24:0/12:1)	$C_{41}H_{83}N_2O_6P$	730.598	15.523	731.605	1.547	0.030	Down
8-Hydroxyquinoline	C_9H_7NO	145.052	8.294	146.059	1.607	0.030	Down
7-Ketodeoxycholic acid	$C_{24}H_{38}O_5$	406.270	12.487	429.259	1.538	0.031	Down
PC (18:4e/2:0)	$C_{28}H_{50}NO_7P$	543.331	14.350	544.338	1.501	0.031	Down
3-amino-2-phenyl-2H-pyrazolo[4,3-c] pyridine-4,6-diol	$C_{12}H_{10}N_4O_2$	242.079	9.928	243.087	1.537	0.031	Down
Deoxycorticosterone	$C_{21}H_{30}O_3$	330.218	11.790	331.226	1.540	0.032	Down
Quinoline-4-carboxylic acid	$C_{10}H_7NO_2$	173.047	8.309	174.054	1.585	0.033	Down
γ-Linolenic acid ethyl ester	$C_{20}H_{34}O_2$	330.276	15.637	331.283	1.504	0.033	Down
PC (16:0e/22:5)	$C_{46}H_{84}NO_7P$	793.591	15.595	794.598	1.577	0.033	Down
β-Cortolone	$C_{21}H_{34}O_5$	348.229	12.894	349.236	1.462	0.035	Down
SM (d14:0/28:1)	$C_{47}H_{95}N_2O_6P$	814.690	14.679	815.698	1.392	0.037	Down
RKK	$C_{18}H_{38}N_8O_4$	215.151	9.264	216.159	1.421	0.038	Down
Dl-3-Hydroxynorvaline	$C_5H_{11}NO_3$	133.074	1.298	116.070	1.478	0.039	Down
Prostaglandin A3	$C_{20}H_{28}O_4$	314.187	13.339	315.194	1.612	0.041	Down
Choline	$C_5H_{13}NO$	103.099	1.219	104.107	1.430	0.041	Down
(S)-Equol	$C_{15}H_{14}O_3$	242.093	10.610	243.100	1.472	0.042	Down
Nicotinamide	$C_6H_6N_2O$	122.048	1.772	123.055	1.441	0.043	Down

SM (d18:2/23:0)	$C_{46}H_{91}N_2O_6P$	798.659	14.623	799.666	1.405	0.044	Down
SM (d20:0/22:2)	$C_{47}H_{93}N_2O_6P$	812.674	12.554	813.681	1.492	0.045	Down
DAG (16:0/18:2)	$C_{37}H_{68}O_5$	609.533	15.331	610.540	1.470	0.045	Down
D-(+)-Proline	$C_5H_9NO_2$	115.063	1.303	116.070	1.435	0.046	Down
octadec-9-ynoic acid	$C_{18}H_{32}O_2$	262.228	15.625	263.235	1.394	0.049	Down
PC (17:0/17:0)	$C_{42}H_{84}NO_8P$	761.590	15.684	762.597	1.480	0.049	Down
Lysopa 16:0	$C_{19}H_{39}O_7P$	410.242	12.916	411.249	1.414	0.050	Down
3-(4-hydroxy-3-methoxyphenyl) propanoic acid	$C_{10}H_{12}O_4$	218.055	9.739	219.062	2.260	0.000	Up
PC (16:0/16:1)	$C_{40}H_{78}NO_8P$	731.545	14.657	732.552	2.153	0.000	Up
SM (d17:0/24:1)	$C_{46}H_{93}N_2O_6P$	400.336	13.656	401.342	2.057	0.000	Up
1-[2-(2,5-dimethyl-1H-pyrrol-1-yl)-4-nitrophenyl]-1H-imidazole	$C_{15}H_{14}N_4O_2$	282.107	8.777	283.114	2.082	0.001	Up
5-Aminoimidazole-4-carboxamide-1-beta-d-ribofuranosyl 5-monophosphate	$C_9H_{15}N_4O_8P$	338.064	12.795	339.071	2.060	0.001	Up
N-(4-fluorophenyl)-N'-(2-piperidinophenyl) urea	$C_{18}H_{20}FN_3O$	351.110	10.986	352.117	1.978	0.001	Up
ACar 16:1	$C_{23}H_{44}NO_4$	397.318	13.135	398.325	1.973	0.002	Up
PC (20:2/20:3)	$C_{48}H_{86}NO_8P$	818.580	13.210	819.587	1.920	0.003	Up
Taurodeoxycholic Acid	$C_{26}H_{45}NO_6S$	481.285	13.148	482.291	1.925	0.003	Up
5,8-dihydroxy-10-methyl-5,8,9,10-tetrahydro-2H-oxecin-2-one	$C_{10}H_{14}O_4$	180.078	9.707	181.085	1.819	0.004	Up
4-(allyloxy)-1,2-dihydroquinolin-2-one	$C_{12}H_{11}NO_2$	201.078	6.513	202.085	1.845	0.004	Up
Tetranor-12(S)-HETE	$C_{16}H_{26}O_3$	288.169	13.842	289.176	1.835	0.006	Up
Tetradecanedioic acid	$C_{14}H_{26}O_4$	258.182	13.028	259.189	1.889	0.006	Up
SM (d26:1/17:0)	$C_{48}H_{97}N_2O_6P$	828.707	14.263	829.715	1.755	0.006	Up
Ethyl oleate	$C_{20}H_{38}O_2$	310.286	13.551	311.293	1.861	0.007	Up
SM (d15:0/19:0)	$C_{39}H_{81}N_2O_6P$	704.581	13.341	705.588	1.846	0.007	Up
Deoxycytidine	$C_9H_{13}N_3O_4$	227.090	1.652	228.096	1.948	0.009	Up
Gamma-Glu-Leu	$C_{11}H_{20}N_2O_5$	260.138	12.973	261.145	1.781	0.012	Up
Histamine	$C_5H_9N_3$	111.080	1.036	112.087	1.599	0.016	Up

Ethyl chrysanthemumate	$C_{12}H_{20}O_2$	196.146	11.807	197.153	1.639	0.020	Up
3-Methylindole	C_9H_9N	131.073	6.564	132.080	1.701	0.022	Up
6-O-(2-Methylbutanoyl)-α-D-glucopyranosyl α-D-glucopyranoside	$C_{17}H_{30}O_{12}$	408.166	12.947	409.174	1.583	0.024	Up
Indole	C_8H_7N	117.057	6.593	118.065	1.675	0.024	Up
DL-Tryptophan	$C_{11}H_{12}N_2O_2$	204.089	6.583	205.097	1.659	0.025	Up
6-Methylquinoline	$C_{10}H_9N$	143.073	6.582	144.080	1.663	0.025	Up
N,N'-di[4-(2,6-dimethylmorpholino)phenyl]thiourea	$C_{25}H_{34}N_4O_2S$	476.222	8.223	477.229	1.612	0.028	Up
XLR11 N-(2-fluoropentyl) isomer	$C_{21}H_{28}FNO$	329.219	8.662	330.226	1.489	0.029	Up
SM (d19:2/17:0)	$C_{41}H_{81}N_2O_6P$	728.581	13.918	729.588	1.519	0.029	Up
PC (16:2e/22:5)	$C_{46}H_{80}NO_7P$	789.564	14.953	790.572	1.607	0.033	Up
PE (14:1e/24:4)	$C_{43}H_{78}NO_7P$	751.550	14.113	752.559	1.541	0.033	Up
Lysopg 18:1	$C_{24}H_{47}O_9P$	510.295	11.976	511.302	1.538	0.034	Up
7Z,10Z,13Z,16Z,19Z-docosapentaenoic acid	$C_{22}H_{34}O_2$	330.255	15.161	331.262	1.647	0.035	Up
PC (20:5/20:5)	$C_{48}H_{76}NO_8P$	825.539	14.082	826.546	1.543	0.035	Up
Methionine sulfoxide	$C_5H_{11}NO_3S$	148.019	1.787	149.027	1.481	0.038	Up
Palmitoyl sphingomyelin	$C_{39}H_{79}N_2O_6P$	702.567	16.915	703.574	1.396	0.039	Up
PC (18:1/18:2)	$C_{44}H_{82}NO_8P$	783.566	14.088	784.573	1.496	0.039	Up
SM (d21:0/20:2)	$C_{46}H_{91}N_2O_6P$	798.657	13.413	799.665	1.464	0.039	Up
9-Oxo-ODE	$C_{18}H_{30}O_3$	294.218	14.155	295.225	1.443	0.040	Up
5-[(10Z)-14-(3,5-dihydroxyphenyl) tetradec-10-en-1-yl] benzene-1,3-diol	$C_{26}H_{36}O_4$	412.258	15.282	413.265	1.465	0.043	Up
5-{[2-(3-pyridinyl) piperidino] sulfonyl}-2,1,3-benzothiadiazole	$C_{16}H_{16}N_4O_2S_2$	360.073	11.587	361.080	1.384	0.046	Up
PC (20:3/20:4)	$C_{48}H_{82}NO_8P$	831.574	16.348	832.580	1.492	0.048	Up
PC (18:4e/22:0)	$C_{48}H_{90}NO_7P$	841.652	16.043	842.659	1.432	0.048	Up
Oxohongdenafil	$C_{25}H_{32}N_6O_4$	480.254	8.763	481.261	1.477	0.048	Up
PC (20:2/20:2)	$C_{48}H_{88}NO_8P$	837.612	16.702	838.618	1.451	0.049	Up

Table S2 The differential metabolites between the model and NF group

Name	Formula	Molecular Weight	RT [min]	m/z	VIP	P-value	Trend
4-phenyl-2-[(3-pyridylamino) methylidene] cyclohexane-1,3-dione	$C_{18}H_{16}N_2O_2$	292.119	11.121	293.126	2.300	0.013	Down
Ecgonine methyl ester	$C_{10}H_{17}NO_3$	199.120	10.497	200.128	2.280	0.014	Down
4-(allyloxy)-1,2-dihydroquinolin-2-one	$C_{12}H_{11}NO_2$	201.078	6.513	202.085	2.070	0.020	Down
Thymine	$C_5H_6N_2O_2$	126.043	5.410	127.050	2.058	0.021	Down
PC (16:2e/2:0)	$C_{26}H_{50}NO_7P$	519.331	14.392	520.339	2.046	0.022	Down
PC (18:0e/22:1)	$C_{48}H_{96}NO_7P$	829.708	15.715	830.715	1.984	0.023	Down
3-hydroxy-2-octylpentanedioic acid	$C_{13}H_{24}O_5$	242.151	11.756	243.158	2.034	0.024	Down
PC (21:2/20:5)	$C_{49}H_{84}NO_8P$	845.588	15.218	846.596	2.129	0.025	Down
PC (16:0/17:0)	$C_{41}H_{82}NO_8P$	747.576	16.132	748.585	2.061	0.025	Down
Deoxycytidine	$C_9H_{13}N_3O_4$	227.090	1.652	228.096	2.220	0.026	Down
SM (d16:3/26:0)	$C_{47}H_{91}N2O_6P$	828.667	16.095	829.676	1.965	0.035	Down
LysoPE 18:2	$C_{23}H_{44}NO_7P$	477.284	10.282	478.291	1.953	0.036	Down
LysoPE 14:0	$C_{19}H_{40}NO_7P$	425.252	14.132	426.260	1.896	0.036	Down
Stiripentol	$C_{14}H_{18}O_3$	216.114	11.650	217.121	1.986	0.038	Down
SM (d30:0/12:0)	$C_{47}H_{97}N_2O_6P$	816.698	15.353	817.705	1.964	0.041	Down
PC (14:0e/22:5)	$C_{44}H_{80}NO_7P$	1531.128	16.535	766.573	2.032	0.043	Down
5-fluoro AB-PINACA N-(4-hydroxyphenyl) metabolite	$C_{18}H_{25}FN_4O_3$	386.174	15.026	387.181	1.894	0.044	Down
Morphine D3	$C_{17}H_{16[2]}H_3NO_3$	288.156	9.936	289.164	1.873	0.049	Down
Theobromine	$C_7H_8N_4O_2$	180.064	6.003	181.071	2.737	0.000	Up
SM (d14:3/22:1)	$C_{41}H_{77}N_2O_6P$	362.275	15.007	725.557	2.352	0.006	Up
Cer-NS (d18:1/24:0)	$C_{42}H_{83}NO_3$	1299.270	16.078	650.642	2.444	0.008	Up
Cer-NS (d18:1/22:0)	$C_{40}H_{79}NO_3$	621.604	15.370	622.610	2.149	0.020	Up
SM (d17:0/24:1)	$C_{46}H_{93}N_2O_6P$	400.336	13.656	401.342	2.086	0.020	Up
GNH	$C_{12}H_{18}N_6O_5$	308.122	11.577	309.130	1.934	0.028	Up

Theophylline	$C_7H_8N_4O_2$	180.064	6.831	181.071	2.048	0.037	Up
PC (20:2/20:3)	$C_{48}H_{86}NO_8P$	818.580	13.210	819.587	1.892	0.038	Up
17α-Ethynyl estradiol	$C_{20}H_{24}O_2$	296.177	12.877	297.184	2.010	0.039	Up
PC (18:5e/22:6)	$C_{48}H_{76}NO_7P$	809.521	16.561	810.527	1.940	0.039	Up
D- (+)-Maltose	$C_{12}H_{22}$O11	364.096	1.279	365.103	1.908	0.041	Up
SM (d15:3/28:2)	$C_{48}H_{89}N_2O_6P$	820.642	14.677	821.650	1.878	0.045	Up

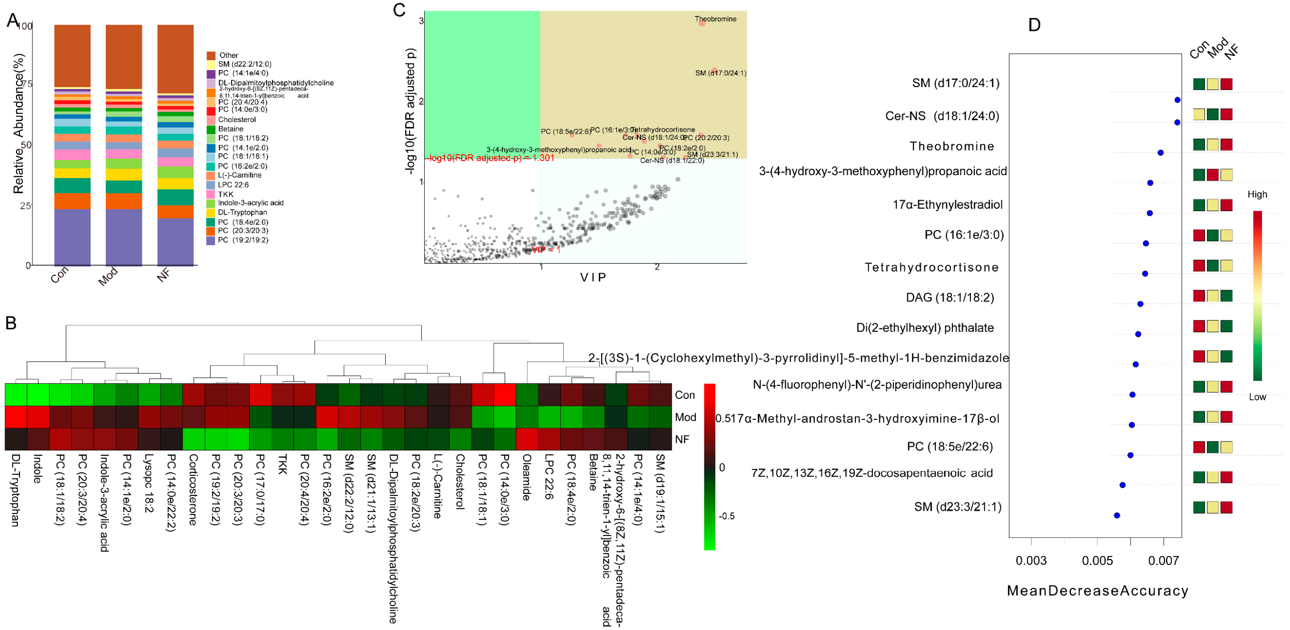

Figure S1. The changes of metabolites of CUMS treated rats after jasmine tea administration. A, stacked column chart of the percentage of metabolites. B, heatmap of the percentage of metabolites. C, Volcano plot of the univariate analysis showing the differentially abundant metabolites based on OPLS-DA analysis. D, Random forest analysis of Mean decrease Accurancy. The right color coding reflected the concentration of this metabolite in different groups. The greater the Mean decrease Accurancy value, the greater their contribution to the sample differentiation.

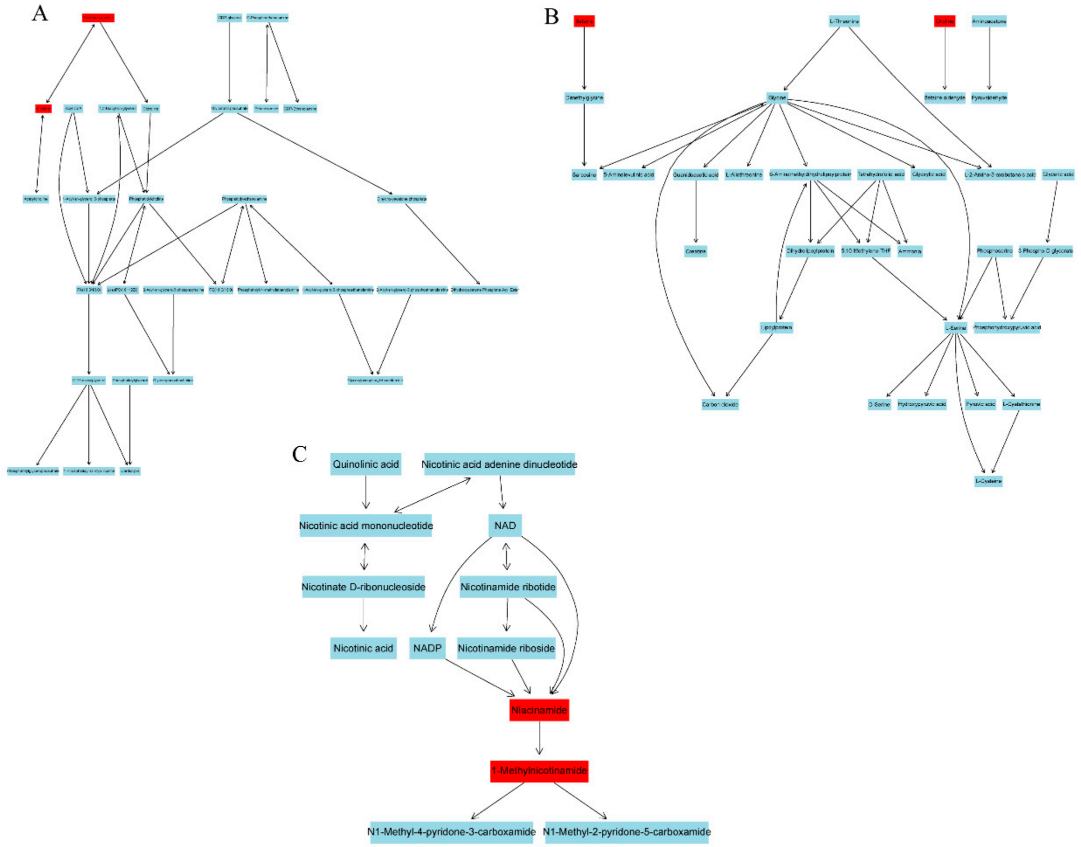

Figure S2. KEGG topological analysis showed that three metabolic pathways (impact value >0, P<0.05) were enriched between the Mod and NF groups. A, Nicotinate and nicotinamide metabolism. B, Glycerophospholipid metabolism. C, Glycine, serine, and threonine metabolism.

A

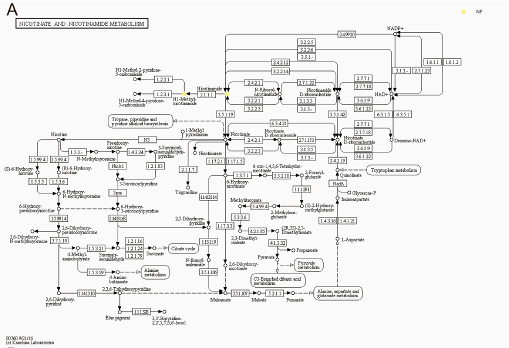
NICOTINATE AND NICOTINAMIDE METABOLISM

B

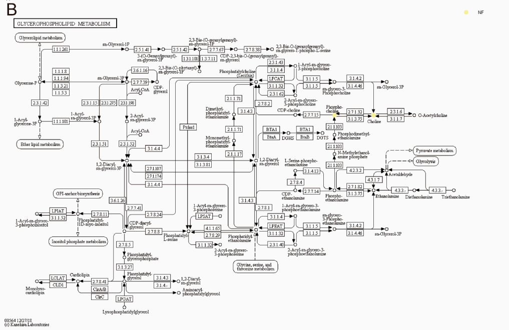
GLYCEROPHOSPHOLIPID METABOLISM

C

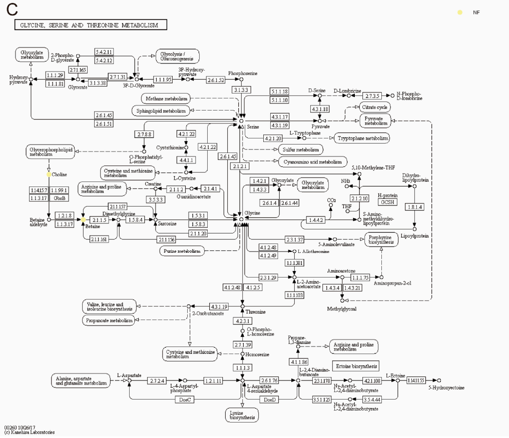
GLYCINE, SERINE AND THREONINE METABOLISM

Figure S3. Metabolic labeling pathway map between the Mod and NF groups. A, Nicotinate and nicotinamide metabolism. B, Glycerophospholipid metabolism. C, Glycine, serine, and threonine metabolism.

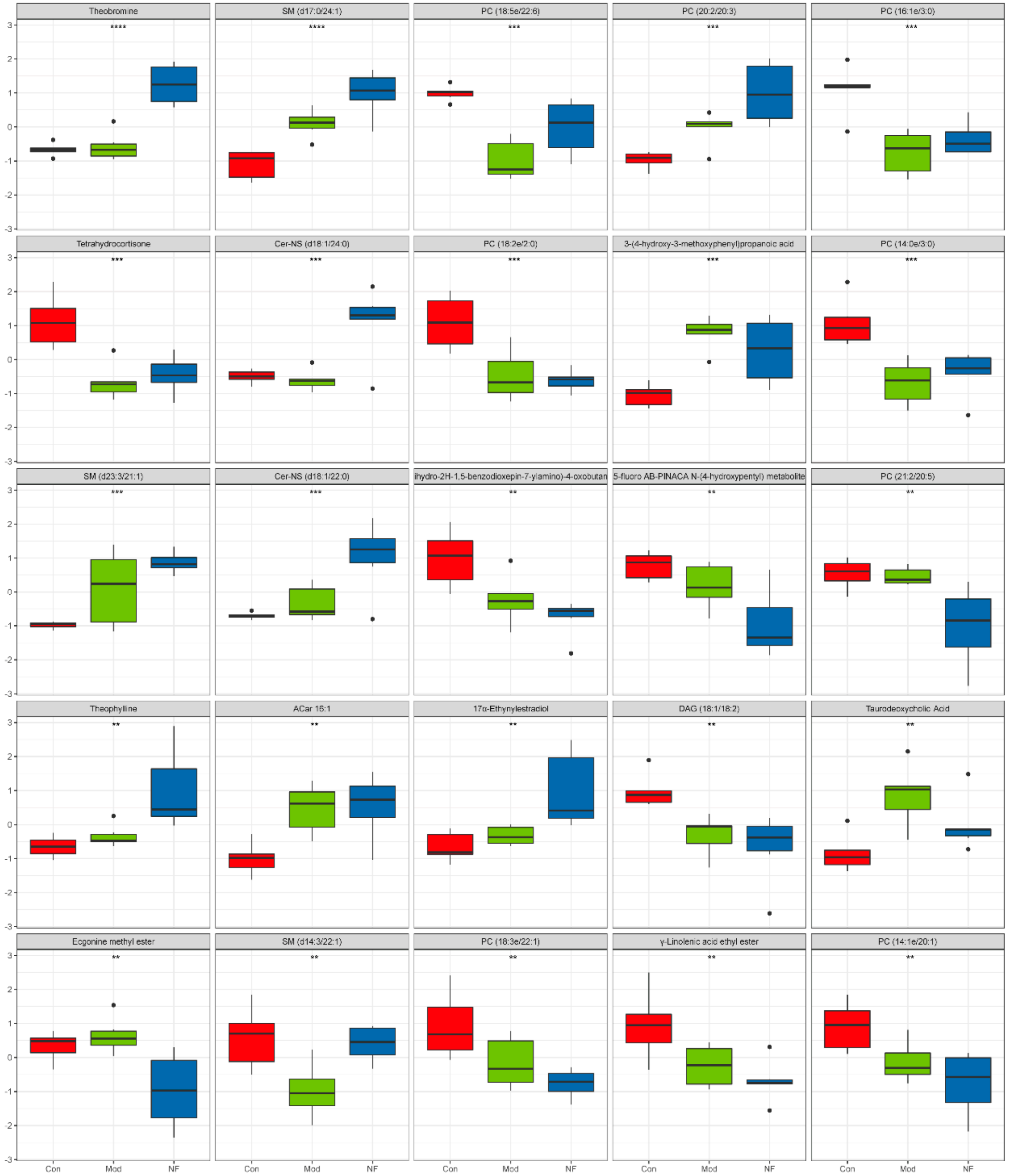
Theobromine
****
SM (d17:0/24:1)
****
PC (18:5e/22:6)
***
PC (20:2/20:3)
***
PC (16:1e/3:0)
***
Tetrahydrocortisone
***
Cer-NS (d18:1/24:0)
***
PC (18:2e/2:0)
***
3-(4-hydroxy-3-methoxyphenyl)propanoic acid
***
PC (14:0e/3:0)
***
SM (d23:3/21:1)
***
Cer-NS (d18:1/22:0)
***
ihydro-2H-1,5-benzodioxepin-7-ylamino)-4-oxobutan
**
5-fluoro AB-PINACA N-(4-hydroxypentyl) metabolite
**
PC (21:2/20:5)
**
Theophylline
**
ACar 16:1
**
17α-Ethynylestradiol
**
DAG (18:1/18:2)
**
Taurodeoxycholic Acid
**
Ecgonine methyl ester
**
SM (d14:3/22:1)
**
PC (18:3e/22:1)
**
γ-Linolenic acid ethyl ester
**
PC (14:1e/20:1)
**
3
2
1
0
-1
-2
-3
Con
Mod
NF

Figure S4. The differential Metabolite among control, model and NF

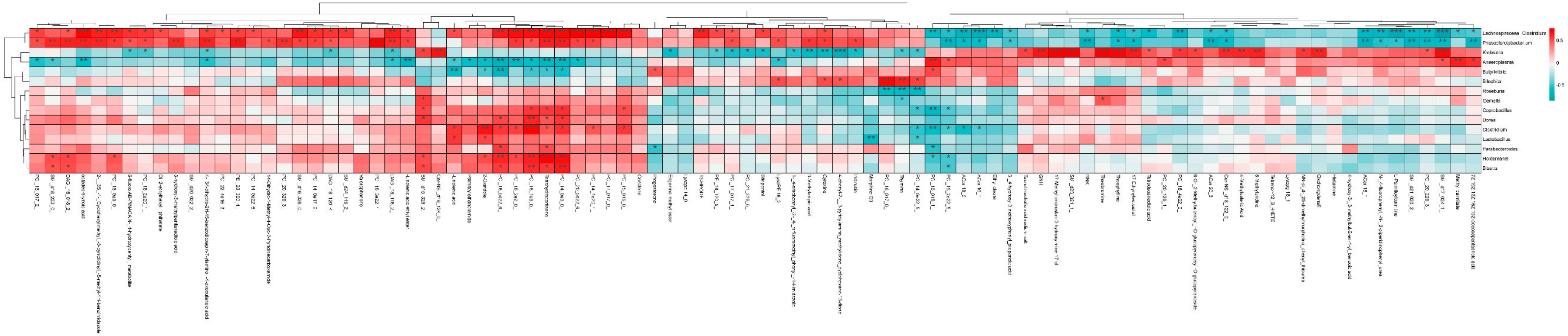

Figure S5. The heatmap of associations of gut based on lefse-genus and metabolites based on OPLS-DA.
